# Supplementary material for: Discordant Humoral and T-Cell Response to mRNA SARS-CoV-2 Vaccine and the Risk of Breakthrough Infections in Women with Breast Cancer, Receiving Cyclin-Dependent Kinase 4 and 6 Inhibitors
Source: Cancers (Basel). 2023 Mar 27;15(7):2000. doi: 10.3390/cancers15072000 (PMC10093435; doi:10.3390/cancers15072000)
Supplement: Supplementary file 1 [file cancers-15-02000-s001.zip › cancers-2141761-supplementary.pdf]

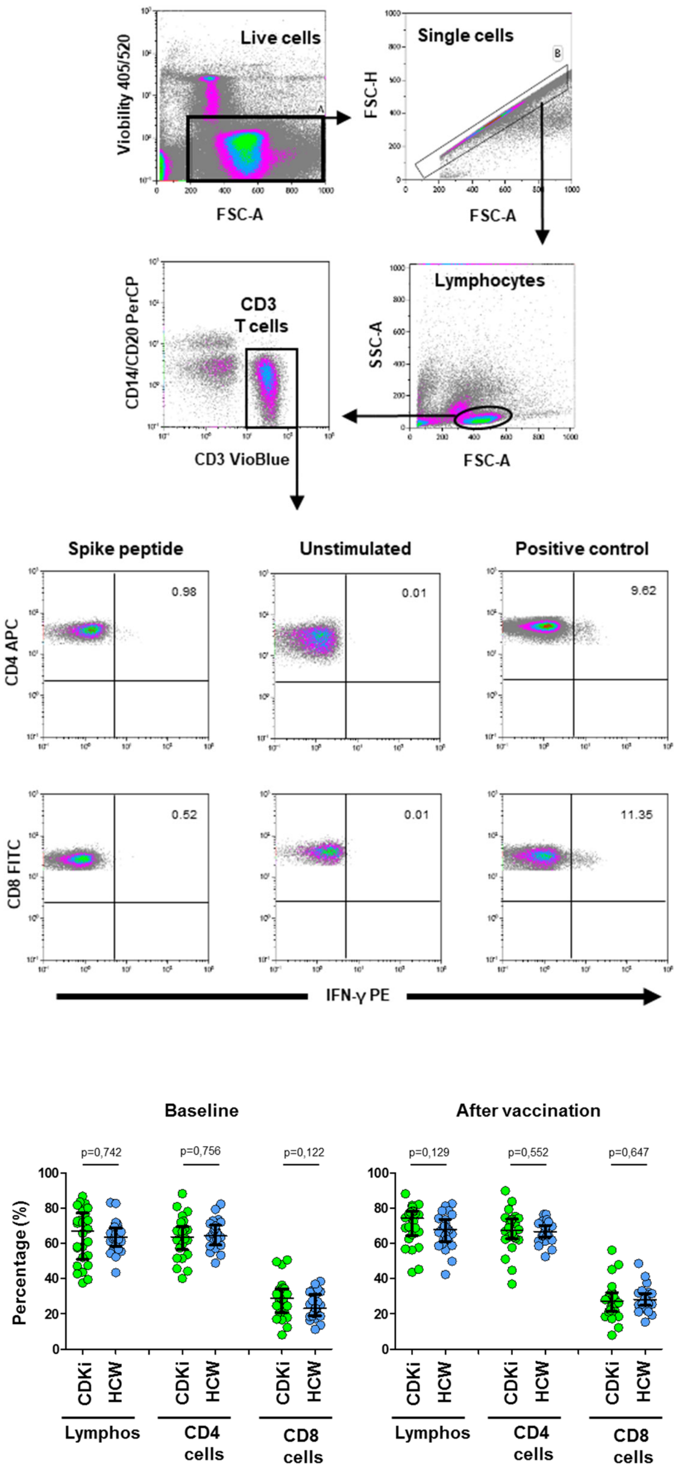

Figure S1: Flow cytometry.

Flow cytometry gating strategy for the quantification of SARS-CoV-2 spike specific T cells in a representative individual. After gating of live cells (Viobility 405/520 / FSC-A) and single cells (FSC-A/FSC-H density plot), lymphocytes were morphologically selected with FSC-A/SSC-A density plot, and then CD3 T cells were gated. Cell debris, monocytes, and B cells were excluded from the analysis with CD14- and CD20-PerCP antibodies. IFN-γ expression was finally analyzed separately for CD4+ and CD8+ T cells and analyzed under three different conditions. Stimulated with SARS-CoV-2 spike peptides, unstimulated (negative control), and SEB-stimulated (positive control). The percentage of cells expressing IFN-γ is shown in each density plot.
